# Rapid treatment of depression with potassium supplementation: relative deficiency of potassium ions and possible pathogenesis—a case report

**DOI:** 10.1186/s13256-025-05693-2

**Published:** 2025-11-28

**Authors:** Jiapei Dai

**Affiliations:** 1 Wuhan Institute for Neuroscience and Neuroengineering, South-Central Minzu University, Minzu Dadao 182, Wuhan, 430074 China; 2 The College of Life Sciences, South-Central Minzu University, Wuhan, 430074 China

**Keywords:** Depression anxiety, Anxiety, Relative deficiency of potassium ions, Potassium supplementation, Case report

## Abstract

**Background:**

Despite advances in depression research and treatment, effective strategies based on clear neural mechanisms are still lacking. Current therapies often show delayed effects and inconsistent outcomes, suggesting that the core pathophysiology remains unclear.

**Case presentation:**

This case report describes a highly successful and rapid resolution of subacute mild anxiety and severe depression in a 32-year-old woman of Chinese origin following treatment with potassium ion supplementation. It explores the potential mechanisms underlying this therapeutic effect, highlighting the role of relative potassium ion deficiency in the pathogenesis of depression.

**Conclusion:**

This case achieved significant therapeutic outcomes through innovative treatment strategies, raising an interesting possibility on the pathogenesis of depression. However, more clinical observations or cohort studies are needed to support this finding.

## Background

Despite ongoing advances in the research and treatment of depression, effective prevention and therapeutic strategies grounded in well-defined neural mechanisms remain lacking. Current treatment approaches are often hindered by delayed onset of action and inconsistent efficacy, indicating that the core pathophysiological processes underlying depression may not yet be fully understood. Previous research has highlighted the critical role of potassium ion homeostasis in regulating neural function [1]. More recent insights propose that a relative deficiency of intracellular potassium ions may contribute to neural dysfunction and the development of neurological disorders [2]. However, the clinical application of this hypothesis has yet to be thoroughly investigated.

## Case presentation

The patient is a 32-year-old married woman of Chinese origin residing in Dongguan city, Guangdong Province, China, born in October 1993, with no children. She reported experiencing recurrent and progressively worsening symptoms of anxiety and depression over the year leading up to April 2022. Specific symptoms included restlessness, low mood, frequent feelings of emptiness and hopelessness, unexplained crying, persistent fatigue, and significant sleep disturbances. Prior to seeking consultation, she had strongly expressed a desire to resign from her position in subway management, a role she had held for over 7 years and once deeply enjoyed.

As a non-blood-related relative of the patient, I am familiar with her upbringing, education, physical health, and work history. She had previously been a lively and emotionally positive individual. Prior to completing her vocational education in 2015, she lived with her parents. After graduation, she began working in subway management in Dongguan, Guangdong Province, China, where she remained employed. According to her parents, they maintained regular video and phone contact with her and had not observed any significant emotional abnormalities. However, her anxiety and depressive symptoms developed gradually over the past year, with a rapid deterioration in April 2022, particularly affecting her interest in work, which was the primary reason for her resignation.

When the patient first informed her parents of her decision to resign, they were deeply surprised. Upon further inquiry, they learned that her resignation was driven by intense negative emotions and severe sleep disturbances during that period. While similar emotional disturbances had occurred in the year leading up to April 2022, they were typically short-lived and tolerable. Both the patient and her husband speculated that these symptoms might be related to the coronavirus disease-19 (COVID-19) pandemic, which led her to neither seek medical diagnosis and treatment nor disclose her condition to her parents or colleagues. However, in April 2022, her symptoms suddenly worsened significantly and became persistent. When her parents asked if there were any specific triggers, she denied any clear connection to family, personal life, or work-related stress. Subsequently, her parents reached out to me, providing details of her emotional changes and seeking my assistance in assessing her condition and offering medical advice.

During our first video call, the patient’s demeanor was strikingly different from what I had expected. She appeared visibly distressed and anxious, frequently breaking into tears—this was in sharp contrast to the cheerful and outgoing lady I had remembered. Her account of her emotional changes matched her parents’ description: over the past year, unexplained anxiety and negative emotions had recurred and intensified, with symptoms becoming particularly severe in the past month. These included a loss of interest in work and daily life, irritability, difficulty concentrating, exhaustion, and sleep disturbances. Even her once-beloved job in subway management now evoked aversion and unease, leading her to submit an oral resignation, citing health reasons. Her supervisors, surprised by her decision given her excellent performance and strong rapport with colleagues, advised her to reconsider and delay her resignation. Instead, they granted her 2 months of paid sick leave to undergo medical evaluations before making a final decision. Based on her self-reported symptoms, a preliminary assessment suggested subacute anxiety and depression, though formal psychological testing was necessary for a definitive diagnosis. Following my recommendation, she underwent a psychological evaluation at a local hospital on 29 April, 2022, which revealed mild anxiety [anxiety severity index based on the self-rating anxiety scale (SAS): 0.59] and severe depression [depression severity index based on the self-rating depression scale (SDS): 0.78]. She then sought my advice regarding treatment options.

After thoroughly analyzing her lifestyle, work, and other factors over the past 3 years—particularly during the 2-year COVID-19 pandemic—I hypothesized that dietary changes might be a significant contributing factor. The long-term consumption of takeout and fast food could have led to a relative potassium deficiency, potentially impairing brain regions involved in emotional regulation and associated neural circuits. Although this was initially speculative, I later developed a systematic theoretical framework and briefly referenced this case in a published paper [2]. However, the author of this report believes that a more detailed, standalone discussion on this topic is warranted.

## Treatment

Given her clinical presentation and individual circumstances, I proposed a tiered treatment strategy as follows:

Standard pharmacotherapy: evidence-based, personalized medication with systematic monitoring.

Nontraditional intervention first: trial of an innovative nonclassical approach (potassium supplementation), switching to conventional medication if ineffective.

Combined therapy: integration of both strategies.

After fully explaining the principles, drug options, and potential outcomes of standard treatment, the patient chose the potassium-focused intervention. The regimen consisted of oral 0.5 g potassium chloride sustained-release tablets after each meal (TID dosing, totaling 1.5 g/day), without adjunct medications.

It should be emphasized that during the treatment period, for which the patient took a paid residential leave, the key lifestyle intervention was the cessation of takeout and fast food. No specialized dependency management or adherence evaluations were conducted. The treatment regimen was successfully implemented without any reported side effects or adverse events.

## Outcome and follow-up

The treatment effect showed a significant time-dependent improvement: initial relief of anxiety and depression was observed in the first week; the trend of symptom improvement continued within the next 2 weeks; by the fourth week, anxiety and severe depression symptoms had disappeared; and by the fifth week, the patient voluntarily requested to resume work. Subsequently, a dynamic potassium supplementation plan was recommended (0.5–1.5 g/day, adjusted as needed), alongside dietary modifications to increase potassium-rich meats. This recommendation was physiologically grounded: animal tissues—particularly excitable cells such as neurons, cardiomyocytes, and muscle cells—maintain intracellular potassium concentrations of approximately 100–400 mM [3, 4]. Minimally processed meats retain this high potassium content, offering superior bioavailability compared with plant-based sources. Over nearly 3 years of follow-up, the patient remained symptom-free, maintaining stable mood health and career performance.

## Discussion

This case illustrates a subacute anxiety-depression episode, with epidemiological and pathophysiological analyses strongly suggesting that chronic potassium deficiency may be a key contributing factor. The findings propose a novel mechanism: relative potassium insufficiency in brain regions involved in emotional regulation may contribute to mood disorders in young adults. This theory warrants exploration from three perspectives: Relative deficiency dynamics: potassium homeostasis and its functions relies on the “intake–distribution–utilization” regulation [1, 5–12]. During periods of insufficient intake, tissue-specific Na^+^-K^+^-ATPase expression creates differential potassium uptake, which could disrupt the function of emotional brain circuits. Susceptibility variability: individuals and tissues differ in their capacity for potassium storage, compensation, and competitive utilization. Some emotional neural networks may be more sensitive to mild or moderate fluctuations in potassium levels [2]. Imbalance mechanism: Na^+^-K^+^-ATPase determines cellular potassium uptake and retention. Under conditions of deficiency, inter-tissue competition may deplete potassium in emotional regulation areas, altering their function. Notably, standard serum potassium tests (3.5–5.5 mM) only reflect blood levels and fail to detect tissue-specific subclinical deficiencies. When deficiency is mild (< 20% below demand), systemic ion regulation may maintain normal serum interval levels while local tissues become deficient, which may explain why individuals with similar potassium deficiencies exhibit varying susceptibilities to various diseases.

## Conclusion

This case achieved significant therapeutic outcomes through innovative treatment strategies, raising an interesting possibility or a new perspective on the pathogenesis of mood illness. However, the applicability of this theory to more complex mood disorders, such as severe depression and bipolar disorder, as well as the causal relationship between changes in potassium ion metabolism and the onset of other systemic diseases, still requires validation through multicenter clinical studies and basic mechanistic experiments. In addition, potassium supplementation should be undertaken with medical supervision.

## Data Availability

All relevant data supporting the findings of this case report are included in the manuscript. Additional data are available from the corresponding author upon reasonable request.
